# Effects of Carrying Police Equipment on Spatiotemporal and Kinetic Gait Parameters in First Year Police Officers

**DOI:** 10.3390/ijerph17165750

**Published:** 2020-08-09

**Authors:** Mario Kasović, Lovro Štefan, Krunoslav Borovec, Martin Zvonař, Jan Cacek

**Affiliations:** 1 Faculty of Kinesiology, University of Zagreb, 10 000 Zagreb, Croatia; mario.kasovic@kif.hr; 2 Faculty of Sports Studies, Masaryk University, 62 500 Brno, Czech Republic; cacek@fsps.muni.cz; 3 Police Academy, Police College, 10 000 Zagreb, Croatia; kborovec@mup.hr; 4 Faculty of Science, Masaryk University, 62 500 Brno, Czech Republic; zvonar@fsps.muni.cz

**Keywords:** load, distribution, walking, force, special population

## Abstract

The main purpose of the study was to explore the effects of carrying police equipment on spatiotemporal and kinetic gait parameters. Two-hundred and seventy-five healthy men and women attending police academy (32% women) were randomly recruited. Gait analysis without and with a police equipment load (≈3.5 kg) was analyzed using the Zebris pressure platform. Differences and effect sizes were calculated using a Student *t*-test and Wilcoxon test for dependent samples and Cohen’s D statistics. In both men and women, carrying police equipment significantly increased the foot rotation (effect size 0.13–0.25), step width (0.13–0.33), step time (0.25), stride time (0.13–0.25) and peak plantar pressure beneath the forefoot (0.16–0.30), midfoot (0.15–0.32) and hindfoot (0.13–0.25) region of the foot. Significant reductions in the step length (0.12–0.25), stride length (0.14–0.23), cadence (0.15–0.28) and walking speed (0.20–0.22) were observed in both sexes. Although significant, the effect sizes were mostly trivial in men and small in women. Our study shows significant changes in the spatiotemporal and kinetic gait parameters when carrying police equipment for both men and women. Although the effect sizes are trivial to small, carrying police equipment of ≈3.5 kg may have a negative impact on gait characteristics in first-year police officers.

## 1. Introduction

Carrying excessive load represents a main physical activity for duty officers, especially police [1]. Police officers are considered as performing at the maximal level, executing highly demanding everyday activities, including running, jumping and carrying heavy objects [2,3]. Such extreme loading conditions may lead to a deviated foot function and structure [4]. The foot is considered an essential part of the body, since it provides the only contact with the ground [4]. Furthermore, it absorbs various shocks over irregular surfaces and maintains forward propulsion [5]. Foot changes, along with weight carriage, often lead to altered kinematic, kinetic and electromyography gait parameters [6].

In the military [7] and police [1,8,9,10,11], gait alternations influenced by load carriage have been less studied. Specifically, a study by Birrell and Haslam [7] has shown that additional load significantly decreases the range of motion of flexion and extension of the knee and pelvic rotation and increases abduction/adduction and pelvis tilt. Another study conducted among active duty police officers has shown a significant decline in the stride length, while no changes in the gait velocity, stride width and cadence were observed [10]. During key occupation tasks, one study has shown a reduced mobility and greater physiological effort when the carried load increased [8]. In general, Joseph et al. [11] showed that the performance of both power and agility was shown to decrease when a tactical load was added to the participants, generating a higher risk of injuries and fatality. Other spatiotemporal parameters, including a slower walking speed and acceleration, have been documented [9].

According to the above, only a handful of studies have examined the potential effects of load carriage on spatiotemporal gait parameters in police officers. By discovering possible effects, health-related professionals (especially podiatrists and orthopedics) and ergonomic designers can re-design the existing police equipment (specifically the belt characteristics), in order to minimize gait deviations.

Therefore, the main purpose of this study was to explore the effects of carrying equipment on spatiotemporal and kinetic gait parameters in a sample of first-year police officers.

## 2. Materials & Methods

### 2.1. Study Participants

The study population consisted of Croatian police academy police officers, who were part of the annual physical fitness testing protocol. Every year, the police academy recruits ≈ 750 men and women in a one-year training program, after which they become a part of the Croatian police system and are qualified to perform police duties and tasks. At the first stage, we randomly recruited 320 men and women currently in the program. After the screening, 23 of them had missing data and 22 did not complete the measurement. Finally, our analyses were based on 275 men and women (mean ± SD age 22 ± 2 years, height 176 ± 9 cm, weight 76 ± 14 kg, body-mass index 24 ± 4 kg/m^2^, 32% women). The data were presented in mean ± SD or otherwise noted. Before the study began, we had given information regarding the study aims, hypotheses, benefits and potential risks. All the procedures were anonymous and in accordance with the Declaration of Helsinki. Additionally, the study was approved by the ethical committee of the Police School ‘Josip Jović’ (Ethical code: 2020). Furthermore, all participants gave written informed consent to participate in the study.

### 2.2. Police Equipment

In the sample we were studying, standard police equipment during police academy consists of several components: (1) belt (≈0.5 kg); (2) a pistol with a full handgun’s magazine (≈1.5 kg); (3) an additional full handgun’s magazine (≈0.5 kg), (4) a nightstick (≈0.8 kg) and (5) handcuffs (≈0.2 kg). In total, the whole equipment without a police suit weighs ≈ 3.5 kg.

### 2.3. Gait Analysis Assessment

To assess the spatiotemporal and kinetic gait parameters, we used a Zebris plantar pressure platform (FDM; GmbH, Munich, Germany; number of sensors: 11.264; sampling rate: 100 Hz; sensor area: 149 cm × 54.2 cm). We followed the methodology of collecting data from previous studies [12,13]. In brief, each participant was instructed to walk at a comfortable speed across the platform without shoes and socks. Additionally, all participants were required to look straight ahead without targeting the pressure platform. They needed to complete five trials across the pressure platform, as recommended by previous evidence [14]. After completing the first task, the same task was repeated while carrying police equipment.

### 2.4. Data Analysis

The basic descriptive statistics are presented as the mean ± SD or median (25th–75th percentile range) for normally and non-normally distributed variables. In order to achieve 80% power to detect a difference in the proportions of spatiotemporal and kinetic parameters in the two groups, where each group (men and women) comprised one fifth of the sample, and where the overall proportion was 0.5 with a two-sided test at the 0.05 level, a total of 250 participants would be needed. Kolmogorov–Smirnov (normality of the distribution) and Levene’s (homogeneity of variance) tests were calculated for all experimental data before inferential testing. Of note, the Kolmogorov–Smirnov test and Leven’s test showed that all the data were normally distributed, except for foot rotation (Kolmogorov–Smirnov statistic for left foot, right foot, left midfoot and right midfoot = 0.09, 0.06, 0.13 and 0.13, *p* < 0.001). Differences between the two conditions (‘no police equipment’ vs. ‘carrying police equipment’) were analyzed using a Student *t*-test and Wilcoxon test for the dependent samples, stratified by sex. To calculate whether differences between sexes (time*sex) occurred, we used a repeated measures analysis of variance. Cohen *d* effect sizes were also calculated to determine the magnitude of the group differences under the two conditions. The effect size was classified as follows: <0.2 was defined as trivial; 0.2–0.6 was defined as small; 0.6–1.2 was defined as moderate; 1.2–2.0 was defined as large; >2.0 was defined as very large; and >4.0 was defined as extremely large [15]. The significance was set at a priori *p* ≤ 0.05. All analyses were performed in Statistical Packages for Social Sciences (SPSS Inc., Chicago, Illinois, USA).

### 2.5. Ethical Statement

This study was approved by the Ethical Committee of the Police School ‘Josip Jović’, Croatia (Ethical code number: 2020).

## 3. Results

The basic descriptive statistics are presented in Table 1. As expected, men were taller, heavier and had higher body-mass index values when compared to women.

Table 2 shows spatiotemporal and kinetic parameter changes during gait in men. The largest effect sizes were observed in the time parameters, followed by the peak plantar pressure and spatial parameters. Although significant, Cohen’s D values showed only trivial-to-small effects when carrying police equipment. Of note, no significant differences between the left and right foot were observed, with the exception of foot rotation (*Z*-test = −5.23, *p* < 0.001). 

Table 3 shows spatiotemporal and kinetic parameter changes during gait in women. Most significant changes were observed in the peak plantar pressure parameters, followed by the spatiotemporal parameters. The effect sizes showed mostly small changes between the two conditions. As was the case for men, no significant differences between the left and right foot were observed, with the exception of foot rotation (*Z*-test = −3.44, *p* < 0.001).

When comparing the magnitude of change between ‘no carrying’ vs. ‘carrying police equipment’ between the sexes, women exhibited somewhat larger effect sizes, especially in terms of peak plantar pressures and spatial parameters, when compared to men. However, no significant time*sex interactions were observed (*p* = 0.068–0.978).

## 4. Discussion

The main purpose of the study was to explore the effects of carrying police equipment on spatiotemporal and kinetic gait parameters in first-year police officers. Our main findings are: (1) after load carriage, both men and women show an increased foot rotation, step width, step time, stride time and peak plantar pressure beneath the forefoot, midfoot and hindfoot region of the foot; (2) a reduction of the values in step length, stride length, cadence and walking speed in both sexes; and (3) although women have larger effect sizes when compared to men, no significant time*sex interactions are observed.

Our findings are partially in line with previous studies conducted among military and police personnel [1,7,8,9,10]. Specifically, among numerous factors studied, a study by Birrell and Haslam [7] conducted among military has shown a significant decrease in the step and stride lengths when a load was added. Although the literature does not provide a clear biomechanical mechanism, one previous study has hypothesized that both the step and stride lengths are shortened in an effort to reduce the modification to walking patterns observed when carrying the load. In this way, the whole load distribution may not be as stressful, especially in the metatarsal bones [16]. Similar changes with a decrease in the step length were observed in active duty police officers [10], with no significant changes in the gait velocity, stride width and cadence, which is inconsistent with our findings. Previous evidence conducted among military students has shown a significant increase in the step width, which is consistent with our results [17]. However, the same study showed no significant gait velocity changes when different load carriages were worn. All studies used a small sample size, which may have underpowered the magnitude of change, which may therefore have been different from our findings. Moreover, in a study by Ramstrand et al. [10], the inclusion criterium required that one be serving as an active duty uniformed officer routinely wearing standard equipment, while we conducted the study among first-year police officers who have only been part of the police academy for six months. Another potential difference is the fact that previous studies used an external load (a vest and belt equipment), while we only used a standardized belt as a load proxy. Furthermore, we used a pressure platform, which generally estimates all spatiotemporal and kinetic parameters based on the number of trials individuals walked across the platform. In a study by Park et al. [17], data were collected for only four steps, while on average our individuals had to walk ten steps (five trials), which increased the level of accuracy. Finally, a study by Bæk et al. [1] has shown no significant differences in cadence, gait velocity, stride length and width. It is speculated that, besides a small sample, participants were all active duty police officers and were older than in our sample, which potentially led to differences in the findings. Although no study to date has investigated the effects of load carriage and foot rotation in police officers, we found significant main effects on the higher outward foot rotation in both legs after carrying police equipment. Previous studies have shown a significant increase in the range of motion of the hip in the frontal and transverse planes as the load increases, pointing out that abduction/adduction and rotation may rotate both feet more outwardly [7]. Unfortunately, we did not collect data related to body kinematics using positional markers, so we can only speculate that a higher range of motion has led to a more pronounced foot rotation.

Our study also shows significant increments in the peak plantar pressures under different foot regions, which is consistent with previous findings based on the military [17]. It has been well-documented that increased load and impact forces under weight-bearing conditions and soldiers’ specific environment often lead to more injuries and the possibility of developing blisters [18]. Additionally, increased impact forces have been shown to be significant predictors of metatarsal stress fractures and joint problems, which often lead to a higher peak plantar pressure [19]. Although we did not collect data related to the foot structure, studies have shown that an excessive load flattens the feet and lowers their arches, often causing pain and discomfort in lower extremities [17]. According to our findings, where the percent changes beneath foot regions increased between 3% and 15% in men and 3% and 17% in women, military boots or special orthotic devices may be components that would be significant in accommodating an increased load and in preventing injury risks.

This study has a few limitations: (1) by using a cross-sectional design, we cannot determine the causality of the correlation, (2) we did not collect 3-D gait analysis data with predefined markers set up on different body parts, so we could not calculate the range of motion in different joints, (3) previous studies have used full police equipment, including a vest, while we only used the load around the belt, which might have led to different magnitudes of change, and (4) we only recruited first-year police officers who were still attending the police academy, and by collecting the data from active duty police officers we may have obtained different spatiotemporal and kinetic gait parameters.

## 5. Conclusions

Our study shows that police load carriage significantly increases foot rotation, step width, step time, stride time and peak plantar pressure beneath the forefoot, midfoot and hindfoot regions of the foot, while decreasing step length, stride length, cadence and walking speed in both men and women. Although the limitations of this study have been well-acknowledged, our findings may add a significant contribution to research on the potentially negative impact of existing police equipment on gait parameters.

## Figures and Tables

**Table 1:** Basic descriptive statistics of the study participants (*N* = 275).

Study Variables	Total (*N* = 275)	Men (*N* = 186)	Women (*N* = 89)	*p* for Sex
	mean ± SD	mean ± SD	mean ± SD	
Age (years)	22 ± 3	22 ± 3	22 ± 3	0.549
Height (cm)	176 ± 9	181 ± 6	166 ± 5	<0.001
Weight (kg)	76 ± 14	83 ± 11	63 ± 8	<0.001
Body-mass index (kg/m^2^)	24 ± 4	25 ± 3	23 ± 2	<0.001

*p* < 0.05.

**Table 2:** Effects of carrying police equipment on spatiotemporal and kinetic gait parameters in men (*N* = 186).

Study Variables	No Police Equipment	Carrying Police Equipment	∆ (%)	Effect Size	*p*-Value
	mean (SD)	mean (SD)			
Foot rotation (°) *					
Left foot	6.8 (3.5–12.0)	7.6 (4.4–12.2)	12.3%	0.13	0.037
Right foot	9.2 (5.9–13.4)	9.6 (6.4–14.7)	9.0%	0.14	0.015
Step length (cm)					
Left foot	68.2 (6.0)	67.5 (6.0)	−1.0%	−0.12	0.027
Right foot	67.8 (6.1)	67.0 (5.8)	−1.2%	−0.13	0.026
Stride length (cm)	136.0 (11.1)	134.5 (10.7)	−1.1%	−0.14	0.007
Step width (cm)	13.5 (3.2)	13.9 (3.1)	3.0%	0.13	0.023
Step time (s)					
Left foot	0.56 (0.04)	0.57 (0.04)	1.2%	0.25	<0.001
Right foot	0.56 (0.04)	0.57 (0.05)	1.2%	0.25	<0.001
Stride time (s)	1.12 (0.08)	1.14 (0.08)	1.2%	0.25	<0.001
Cadence (steps/min)	107.2 (6.7)	106.2 (7.0)	−0.9%	−0.15	0.002
Speed (km/h)	4.4 (0.5)	4.3 (0.5)	−2.3%	−0.20	0.003
Peak pressure (N/cm^2^)					
(a) Forefoot					
Left foot	47.1 (13.0)	49.5 (11.7)	5.1%	0.19	0.004
Right foot	48.3 (11.7)	50.2 (12.3)	3.9%	0.16	0.014
(b) Midfoot *					
Left foot	14.2 (10.6–22.3)	16.1 (11.8–21.8)	8.2%	0.15	0.023
Right foot	14.2 (10.8–19.3)	16.5 (12.9–21.9)	15.0%	0.26	<0.001
(c) Hindfoot					
Left foot	35.3 (8.6)	37.0 (9.3)	4.8%	0.19	<0.001
Right foot	33.7 (8.1)	34.8 (9.3)	3.3%	0.13	0.038

* denotes using median (interquartile range). ∆ (%)—percent changes between ‘No police equipment’ and ‘Carrying police equipment’ measurements. *p* < 0.05.

**Table 3:** Effects of carrying police equipment on spatiotemporal and kinetic gait parameters in women (*N* = 89).

Study Variables	No Police Equipment	Carrying Police Equipment	∆ (%)	Effect Size	*p*-Value
	mean (SD)	mean (SD)			
Foot rotation (°) *					
Left foot	4.0 (2.0–5.9)	5.0 (2.5–7.0)	38.3%	0.23	0.034
Right foot	5.7 (2.7–8.3)	6.4 (2.5–9.7)	26.5%	0.25	0.002
**Step length (cm)**					
Left foot	66.7 (5.4)	65.7 (5.0)	−1.5%	−0.19	0.020
Right foot	67.0 (5.3)	65.7 (5.0)	−1.9%	−0.25	0.004
**Stride length (cm)**	133.7 (9.8)	131.4 (8.9)	−1.7%	−0.23	0.003
**Step width (cm)**	9.8 (2.5)	10.6 (2.4)	8.2%	0.33	0.002
**Step time (s)**					
Left foot	0.53 (0.04)	0.54 (0.04)	1.9%	0.25	<0.001
Right foot	0.53 (0.04)	0.54 (0.04)	1.9%	0.25	0.036
**Stride time (s)**	1.06 (0.07)	1.07 (0.08)	0.9%	0.13	<0.001
**Cadence (steps/min)**	112.3 (13.2)	109.3 (7.3)	−2.7%	−0.28	0.034
**Speed (km/h)**	4.6 (0.5)	4.5 (0.4)	−2.2%	−0.22	0.005
**Peak pressure (N/cm^2^)**					
**(a) Forefoot**					
Left foot	45.0 (11.3)	48.3 (10.9)	7.3%	0.30	0.005
Right foot	46.1 (12.9)	48.9 (11.9)	6.1%	0.23	0.009
(b) Midfoot *					
Left foot	11.9 (9.2–16.5)	13.8 (10.8–19.9)	16.8%	0.32	0.020
Right foot	12.1 (8.8–16.2)	14.6 (9.9–18.6)	14.7%	0.30	0.033
**(c) Hindfoot**					
Left foot	34.6 (8.5)	36.4 (8.9)	5.2%	0.21	0.038
Right foot	34.4 (8.2)	36.5 (8.6)	6.1%	0.25	0.011

* denotes using median (interquartile range). ∆ (%)—percent changes between ‘No police equipment’ and ‘Carrying police equipment’ measurements. *p* < 0.05.
